# Mental Health Disorders Due to Gut Microbiome Alteration and NLRP3 Inflammasome Activation After Spinal Cord Injury: Molecular Mechanisms, Promising Treatments, and Aids from Artificial Intelligence

**DOI:** 10.3390/brainsci15020197

**Published:** 2025-02-14

**Authors:** Pranav Kalaga, Swapan K. Ray

**Affiliations:** Department of Pathology, Microbiology, and Immunology, University of South Carolina School of Medicine, 6439 Garners Ferry Road, Columbia, SC 29209, USA; pkalaga@email.sc.edu

**Keywords:** spinal cord injury, gut dysbiosis (GD), gut microbiome, gut-brain axis (GBA), NLRP3 inflammasome, pro-inflammatory cytokines, mental health disorders, artificial intelligence (AI)

## Abstract

Aside from its immediate traumatic effects, spinal cord injury (SCI) presents multiple secondary complications that can be harmful to those who have been affected by SCI. Among these secondary effects, gut dysbiosis (GD) and the activation of the NOD (nucleotide-binding oligomerization domain) like receptor-family pyrin-domain-containing three (NLRP3) inflammasome are of special interest for their roles in impacting mental health. Studies have found that the state of the gut microbiome is thrown into disarray after SCI, providing a chance for GD to occur. Metabolites such as short-chain fatty acids (SCFAs) and a variety of neurotransmitters produced by the gut microbiome are hampered by GD. This disrupts healthy cognitive processes and opens the door for SCI patients to be impacted by mental health disorders. Additionally, some studies have found an increased presence and activation of the NLRP3 inflammasome and its respective parts in SCI patients. Preclinical and clinical studies have shown that NLRP3 inflammasome plays a key role in the maturation of pro-inflammatory cytokines that can initiate and eventually aggravate mental health disorders after SCI. In addition to the mechanisms of GD and the NLRP3 inflammasome in intensifying mental health disorders after SCI, this review article further focuses on three promising treatments: fecal microbiome transplants, phytochemicals, and melatonin. Studies have found these treatments to be effective in combating the pathogenic mechanisms of GD and NLRP3 inflammasome, as well as alleviating the symptoms these complications may have on mental health. Another area of focus of this review article is exploring how artificial intelligence (AI) can be used to support treatments. AI models have already been developed to track changes in the gut microbiome, simulate drug-gut interactions, and design novel anti-NLRP3 inflammasome peptides. While these are promising, further research into the applications of AI for the treatment of mental health disorders in SCI is needed.

## 1. Introduction

Spinal cord injury (SCI) is a serious neurological disorder in the central nervous system (CNS), and it is estimated to impact 25 to 59 people per million in the United States of America (USA) each year [1]. A recent study investigated the epidemiology of SCI and associated mortality in the USA over the past decade (2010–2021), showing these outcomes: the incidence of SCI continued to increase, cervical and incomplete injuries kept increasing, complete injuries declined, and 1-year mortality decreased [2]. Amongst the many reasons for SCI, the leading causes are falls, motor vehicle accidents, and other incidental injuries [3]. The pathogenesis of SCI is complex, with many stages following the primary injury [4]. Common forms of secondary damage after SCI include hemorrhaging, demyelination, extreme glutamate release, and free radical formation, to name a few [5]. The focus of this article will be on gut dysbiosis (GD) resulting from an imbalance in the gut microbiome (also called gut microbiota) and inflammasome activation, the two forms of secondary detrimental mechanisms post SCI that can exacerbate the mental health disorders in the SCI patients. A study by the Royal Australian and New Zealand College of Psychiatrists found that nearly half the SCI patients suffered from at least one form of mental health disorder, such as depression (37%), anxiety (30%), clinically legitimate stress (25%), or post-traumatic stress disorder (PTSD, 8.4%) [6]. Overall, SCI patients showed a 2-fold or more increase in mental health disorders when they were compared to the general population. Of the SCI patients with one mental health disorder, 60% also suffered from at least one other emotional disorder, indicating a genuine 56% increase over the common population. Given how prevalent the post SCI mental health disorders are, it is crucial to gain a thorough understanding of the mechanisms that cause the intensification of these disorders in the aftermath of SCI. This knowledge will eventually help us devise better treatments for SCI-associated mental health disorders using novel therapeutic agents and aids from artificial intelligence (AI).

**Figure 1:**
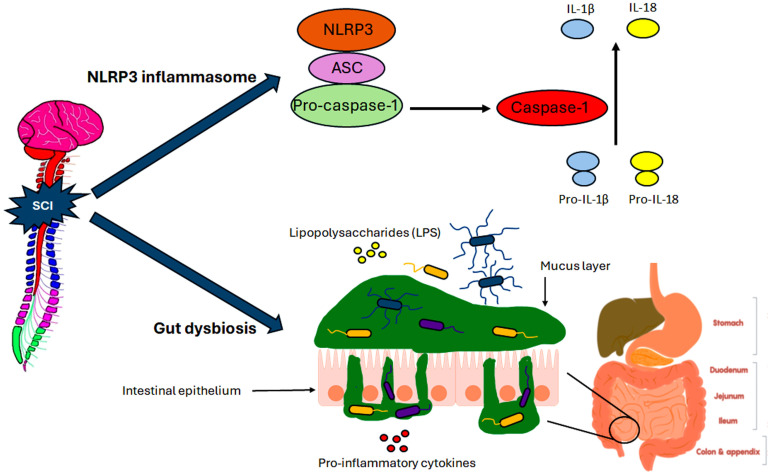
GD occurrence and NLRP3 inflammasome activation after SCI. Two common secondary consequences in the aftermath of SCI are the occurrence of GD and activation of the NLRP3 inflammasome. The NLRP3 inflammasome is comprised of three parts. In the third part, caspase-1 is responsible for transforming pro-IL-1β and pro-IL-18 into corresponding mature cytokines that have an impact on promotion of neuroinflammation. In GD, the mucus layer of the intestinal lumen seeps into the intestinal epithelium, enabling harmful bacterial translocation and imbalance in the gut.

The state of the gut microbiome is relevant to mental health as it has been noted to affect major depressive disorders and schizophrenia [7]. The human gut microbiome is estimated to consist of 300 to 500 diverse types of bacteria that are influenced by factors such as diet, age, and health status [8]. GD, as mentioned earlier, is an imbalance in the bacterial composition of the gut, and it offers a ‘window of opportunity’ for fixing the triggers caused by some complications [9]. In this case, SCI is the trigger of GD, causing a notable change in gut bacterial composition (Figure 1) [10]. Studies show that development of GD is directly due to induction of SCI, and one such study has examined mice with induction of SCI either at T4 or T10 [11]. Compared to the sham control group, both SCI groups of mice exhibited increases in GD [11]. More specifically, testing found an increased level of bacteria from the class Clostridia, which is found to inhibit motor function and recovery from SCI [11]. The other area of interest that can impact mental health post SCI is inflammasome activation. Inflammasomes are large pro-inflammatory protein complexes that can be triggered by stimuli such as pathogens and extracellular ATP released by cell death [12]. Studies in mice SCI models found increasing levels of activation of the NOD (nucleotide-binding oligomerization domain) like receptor-family pyrin-domain-containing three (NLRP3) inflammasome and its signaling proteins post SCI (Figure 1) [13]. NLRP3 is also known as NALP3 (nucleotide-binding-domain, leucine-rich repeat-containing protein 3) or cryopyrin.

Further linkage between GD and mental health disorders has been noted. Studies in rat models have identified changes in gut microbial composition as an indicator of depressive behavior [14]. In humans as well as in animal models with SCI, depressive behavior has been linked with an increase in NLRP3 inflammasome activation [15]. Additionally, the activation of the NLRP3 inflammasome has been implicated in the progression of depression in mice models [16]. Despite the complexity of GD occurrence and NLRP3 inflammasome activation, treatments are being developed to target GD and this inflammasome activation as a method of treating mental health disorders post SCI. Additionally, various developments in the field of AI are opening new possibilities for the treatment of GD and NLRP3 inflammasome activation for mitigation of mental health disorders in SCI.

## 2. GD Due to SCI and Damaging Consequences in the Body

The balanced state of the gut microbiome is essential for the normal functioning of the human body. Although there are many local effects, the activities of the gut microbiome have a significant impact on the functioning of the human body as a whole [17]. Diseases such as irritable bowel syndrome, type 2 diabetes, atopic eczema, and allergies have been linked to improper functioning of the gut [18]. Another area of importance is understanding the gut-brain axis (GBA). The GBA functions as a multidirectional path of communication between the brain, spinal cord, enteric system, autonomic system, and hypothalamic pituitary adrenal axis [19]. The gut is key to the functioning of the CNS as it produces different neurotransmitters, including serotonin, which is also called 5-hydroxytryptamine (5-HT), and dopamine (DA) and their precursors, aiding the nervous system in communicating with the entire body [20]. For example, it is estimated that 90% of 5-HT and 50% of DA in the body come from the gut [21]. The gut is not just localized to what are considered gastrointestinal organs but, instead, works as a complex organ that influences almost all processes across the body.

**Figure 2:**
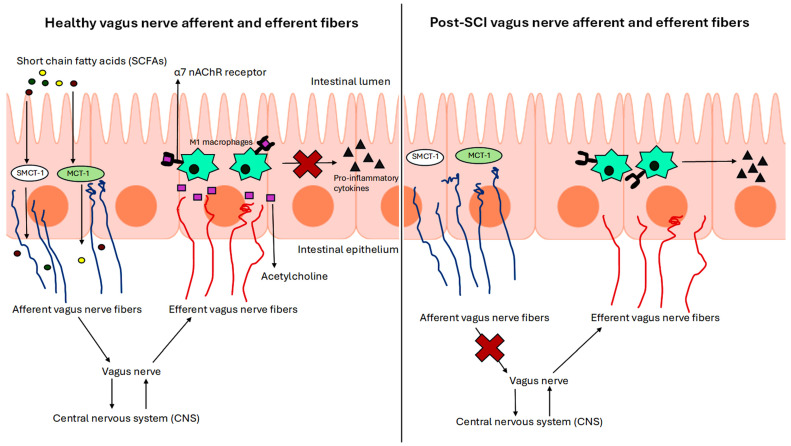
The effects of healthy and damaged vagus nerve fibers on pro-inflammatory cytokine production post SCI. When vagus nerve afferent fibers are functioning normally, they pick up SCFAs and transport them to the CNS via the vagus nerve. After receiving the SCFAs, the CNS sends acetylcholine to the intestines via efferent fibers. Acetylcholine attaches to the alpha7 nicotinic acetylcholine receptor (α7 nAChR) on M1 macrophages (the activated macrophages for inducing pro-inflammatory response) to inhibit the production of pro-inflammatory cytokines. However, when the vagus nerve afferent fibers are damaged due to SCI, this channel of communication is broken, and the CNS does not send any acetylcholine to inhibit the production of pro-inflammatory cytokines. Additionally, there are reduced levels of SCFAs in the intestines after SCI, also negatively impacting this process.

Studies are varied on how bacterial composition in the gut changes post SCI, with changes depending on which vertebrae are injured. Despite this variability, the danger of an imbalanced gut microbial composition persists after the SCI [22]. It is reported that bowel problems occur in 27 to 62% of SCI patients, and approximately 11% of the patients get readmitted for gastrointestinal problems [23,24]. The condition of the spinal cord after an injury is disrupted, and the vagus nerve, along with associated sympathetic nerve fibers of the enteric system, is equally impacted (Figure 2) [25]. These sympathetic nerve fibers often travel alongside arteries to enter the mucosa of the gastrointestinal (GI) tract [26]. These sympathetic nerve fibers serve as communicators between the autonomic nervous system and the mucosa of the GI tract. Changes to this line of communication have a direct impact on the size and quality of the mucosa, where most of the GI-based bacteria live [27]. One study took mucosal biopsies of both the right and left colon of the SCI patients and found significant decreases in neurotransmitters such as acetylcholine and 5-HT in the mucosal biopsies of the SCI patients [28].

Another damaging consequence of the SCI-associated GD is the diminished production of short-chain fatty acids (SCFAs). SCFAs are the products of bacterial fermentation of carbohydrates in the gut microbiome. Common SCFAs include acetate, propionate, and butyrate. These SCFAs are crucial to multiple functions, such as protecting the gut membrane and serving as an energy source [29]. One study found a significant decrease in the number of bacteria of the genera *Faecalibacterium*, *Agathobacter*, and *Megamonas* in human stools following SCI [30]. All these three genera engage in the production of useful SCFAs. If the production of SCFAs is limited due to GD post SCI, it poses plenty of problems to the gut. SCFAs, especially butyrate, work alongside intestinal epithelial cells (IECs) to form a protective barrier by functioning as signaling molecules for the genes that encode barrier components [31].

After a serious trauma like SCI, the intestinal membrane is at a greater risk of increased permeability and bacterial translocation [32]. Interestingly, in a study in mice after stroke, researchers found increased levels of bacteria in the lungs [33]. The study was able to trace these bacteria back to the small intestine through a gut permeability assay and 16S rRNA gene amplicon sequencing and bioinformatics analyses of the tissue from the lungs. Since stroke is a very impactful event on the sympathetic nervous system, this experiment laid the foundation for the idea that there is a capacity for bacterial translocation after an injury via the disruption of sympathetic nerves in the gut [34]. This has opened the door for researchers to explore how GD caused by SCI can influence the gut bacterial composition and functioning of the gut after the injury.

## 3. Inflammasomes and NLRP3 Inflammasome Activation in SCI

Inflammasomes are intracellular multiprotein complexes that include a sensor molecule, an adaptor molecule called apoptosis-associated speck-like protein (ASC), and caspase-1. While there are many distinct types of inflammasomes, the most thoroughly studied one is the NLRP3 inflammasome [35]. The NLRP3 inflammasome is primed by the stimuli from the most prominent pro-inflammatory transcription factor called nuclear factor-kappa B (NF-κB) and the pro-inflammatory cytokines like interleukin-1 beta (IL-1β) that are produced or activated via the gram-negative bacteria derived lipopolysaccharides (LPS) and the toll-like receptor (TLR) pathway. After priming, NLRP3 inflammasome is ready for activation, and the three individual parts work together to make the inflammasome complex. Although the exact mechanism of NLRP3 inflammasome activation is still being investigated, it is believed that activation is triggered by the sensation of a common event from stimuli rather than binding to stimuli [36]. Stimuli that have been noted to cause NLRP3 inflammasome activation include ion fluctuations, reactive oxygen species (ROS), and lysosome damage [37]. Following activation, caspase-1 induces maturation of the pro-inflammatory cytokines, which then start pyroptosis, which is now a well-known form of cell death usually triggered by NLRP3 inflammasome activation [38].

One of the main stimuli for NLRP3 inflammasome activation is ion fluctuations. Studies in the cat model of SCI showed that as soon as one hour after SCI there was a significant efflux of K^+^ from the injury site and significant influx of Ca^2+^ into the injury site [39]. Although there is no conclusive mechanism for why K^+^ efflux post SCI can induce NLRP3 inflammasome activation, there are some theories. One theory is that while K^+^ does not directly block NLRP3, the efflux of K^+^ allows the NIMA (Never In Mitosis gene A)-related kinase 7 (NEK7), which is the smallest serine/threonine kinase in the mammalian NEK family, to bind to NLRP3 [40]. Studies in mouse model of SCI have found that NEK7 promotes NLRP3 signaling [41]. Another theory is that the efflux of K^+^ affects ionic homeostasis, triggering the production of ROS in the mitochondria [40]. Another ion fluctuation that can influence NLRP3 inflammasome activation is the influx of Ca^2+^ into the cells. Excessive intracellular Ca^2+^ is sensed by two G-protein coupled receptors namely Ca-sensing receptor (CaSR) and G protein-coupled receptor family C group 6 member A (GPRC6A) [42]. One study used the small interfering RNAs (siRNAs) to knock down the CaSR gene in mouse model of SCI and found a significant decrease in the pro-inflammatory cytokine IL-1β despite the presence of increased Ca^2+^ concentration, indicating that NLRP3 complex was unable to form. Without CaSR, Ca^2+^ is unable to trigger NLRP3 inflammasome activation [43]. A similar study was conducted with a knockout of the GPRC6A gene and noted the same results, such as reduced amount of IL-1β despite the presence of NLRP3 activators. This suggests that GPRCA6 is also key to the upregulation of NLRP3 via Ca^2+^ influx [44].

Another key stimulus for the activation of NLRP3 inflammasome post SCI is ROS. After SCI, the mitochondria are flooded with an influx of Ca^2+^ ions, causing a signaling loop that results in increased production of ROS. Additionally, the endoplasmic reticulum is disturbed following SCI and is more likely to produce misfolded proteins [45]. Specifically, misfolded disulfide bonds are very potent in triggering the unfolded protein response, which leads to increased production of ROS in the endoplasmic reticulum [46]. A study in Sprague-Dawley rats showed that treatment with rutin, a flavonoid that blocks the production of ROS, significantly decreased ROS levels and subsequent NLRP3 inflammasome activation [47]. A third stimulus that activates NLRP3 inflammasome post SCI is lysosome damage. Studies in rat models have found that in one h after SCI, there is drastic decrease in the lysosomal enzymes such as cathepsin D (CTSD) and N-acetyl-β-glucosaminidase (NAG), both of which are important to lysosome functioning. Given how early the decreases in CTSD and NAG were noted, lysosome damage might be one of the earliest triggers for secondary damages with the activation of NLRP3 inflammasome [48].

One study in rat models found increased levels of NLRP3, ASC, and caspase-1 in 72 h following SCI [49]. Additionally, the study also found that the pro-inflammatory cytokine IL-1β was significantly upregulated during the 72-h period post SCI. Since immune response is generally over reactive in this period post SCI, there is a high chance that the extreme upregulation of pro-inflammatory cytokines like IL-1β propagates excessive immune response meddling by macrophages, microglia, and neutrophils [50]. With the use of Bay 11-7082 (an inhibitor of NF-κB) and A438079 (an antagonist of the purinergic P2X7 receptor, which specifically binds to the purine derivate ATP), researchers evaluated how blocking NLRP3 inflammasome would improve the outcomes of SCI recovery in mice [13]. They found that blocking NLRP3 with these two drugs resulted in decreased infiltration of macrophages, microglia, and neutrophils compared to the control group during the 72 h post SCI. Moreover, researchers noted less cytokine maturation due to the decreased levels of caspase-1.

## 4. GD and Immune System Work Hand-in-Hand for Initiating Pro-Inflammatory Cascade in SCI

It is important to consider how the GD and immune system can influence each other when studying the state of the body and mind post SCI. It has been well established that there is some relationship between the condition of the gut and the immune system in studies with animal models. It was found that decreased complexity in gut bacterial composition hindered the microglial function in immune response [51]. Additionally, mice with no commensal gut bacteria turned up the features of weakened immune system problems such as malformed spleens and lymph nodes, improper B-cell and T-cell zones, and reduced expression of the genes in the IEC layer [52,53,54].

**Figure 3:**
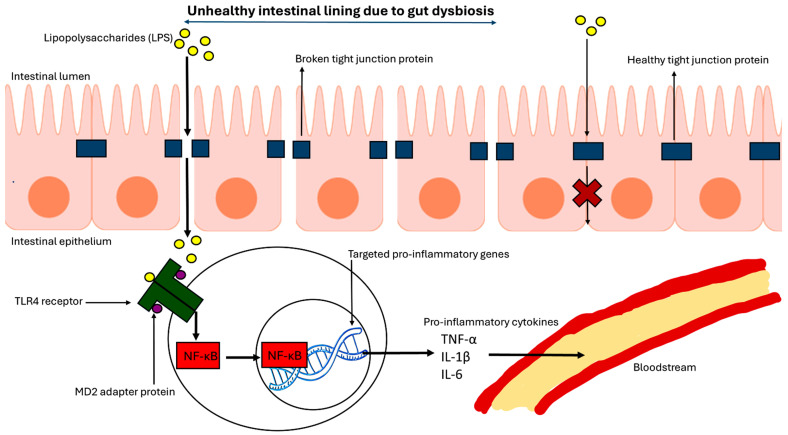
GD releases and enables LPS to activate a signal cascade that produces the pro-inflammatory cytokines. GD damages the tight-junction proteins that otherwise prevent LPS from entering the IEC layer. Once in this layer, LPS can bind to the TLR4 that holds together the MD2 adapter protein in the homodimer complex LPS-MD2-TLR4. From there, a signal cascade is initiated and propagated ending in the production of the powerful pro-inflammatory transcription factor NF-κB. NF-κB binds to the promoter regions of the pro-inflammatory genes in the nucleus and promotes the production of the mRNA molecules that eventually code for production of pro-inflammatory cytokines. Additionally, after these pro-inflammatory cytokines are produced, they have easy access to the bloodstream and can travel throughout the body to spread the inflammatory response.

Another example in which the gut modulates the immune system is the increased prevalence of LPS, derived from harmful gram-negative gut bacteria, following alterations in gut bacterial composition causing GD [55]. Normally, LPS are amphiphilic molecules that are incapable of crossing the intestinal barrier [56]. However, changes in bacterial composition due to GD can weaken tight-junction proteins, which otherwise protect the intestinal barrier, allowing LPS and other detrimental molecules derived from GD to move freely in and out of the gut [57]. LPS can play a significant role in the TLR pathway, acting with the adapter myeloid differentiation protein 2 (MD2) to start the pro-inflammatory signaling cascade (Figure 3). The binding of LPS, MD2, and TLR4 together with high affinity, triggers the formation of the active homodimer LPS-MD2-TLR4 [58]. MD2 readily recognizes the Lipid A domain of LPS for the activation of subsequent signaling cascade for production of cytokines and chemokines, initiating the inflammatory and immune responses. As a result of activation of this pathway, highly potent pro-inflammatory molecules such as NF-κB, IL-1β, TNF-α, and IL-6 are excessively produced [59]. Furthermore, studies in a human blood-brain barrier (BBB) model showed that these pro-inflammatory cytokines were able to pass through the BBB and modulate the brain, triggering apoptotic cell death for promotion of pathophysiology in the brain and detrimental effects in the mental health status [60].

Additionally, there are studies on how GD and NLRP3 inflammasome can interact with each other. Production of NLRP3 inflammasome is triggered by the TLR pathway, which is activated by the byproducts of GD [61,62]. Another study focused on rats, which were exposed to chronic unpredictable mild stress stimuli, found that if control rats were exposed to bacteria from stressed rats via fecal microbiome transplantation (FMT), the control rats would show higher levels of NLRP3 inflammasome activation [63]. The study also tracked the gut bacterial composition of the control and stressed rats, finding the changes in the gut bacterial composition of both groups to be consistent with each other. Furthermore, some of the SCFAs produced in the gut are known to inhibit pro-inflammatory responses [64]. In general, SCFAs inhibit epigenome-modifying enzymes, namely histone deacetylases (HDACs), which are responsible for decreasing the transcription of the genes that code for production of the proteins to control pro-inflammatory signaling pathways [65]. Studies in human Caco-2, a colorectal adenocarcinoma cell line, show that SCFAs limit the production of LPS, leading to a decrease in NLRP3 inflammasome activation [66]. Obviously, there is a connection between the state of gut health and NLRP3 inflammasome functioning. Further research on this connection would prove useful in understanding how this connection could be modified to improve outcomes of mental health disorders in SCI patients.

## 5. GD and Mental Health Disorders

There are multiple studies establishing a link between GD and the prevalence of mental health disorders. In a study with rats, researchers took gut microbiome from depressed animals and transplanted it into germ-free rats [14]. After transplantation, the rats with the transplanted microbiome reported higher levels of anxiety-like behavior, reduced open field activity, and decreased sucrose intake when they were compared to the control rats. In a similar study with humans who had a major depressive disorder (MDD), researchers were able to find significant alterations in gut microbiome from their fecal samples [67]. Specifically, the bacteria from the phyla Bacteroidetes and Proteobacteria in the MDD groups significantly increased when they were compared to the control groups while bacteria from the phylum Firmicutes in the MDD group were significantly decreased when they were compared to the control group. Another study of human fecal samples also reported an increase in the phylum Bacteroidetes from patients with MDD [68]. Specifically, researchers have examined bacteria from the genus *Bacteroides*, which is in the phylum Bacteroidetes, and they have found a significant presence of bacteria from this genus, which has been implicated in the activation of obesity, autoimmune diseases, and cognitive impairments. In addition to the change in the composition of the gut microbiome, GD also impacts the products such as neurotransmitters, amino acids, and SCFAs generated in the gut. Reduced production of these gut metabolites can pretense negative consequences for mental health wellbeing [69].

In patients with MDD, the SCFA producing bacteria have been found to be at lower levels (Table 1). One study comparing the fecal samples of humans with and without MDD found decreased levels of bacteria from the genus *Faecalibacterium* in patients with MDD [70]. Since *Faecalibacterium* is one of the genera of bacteria that produce SCFAs through carbohydrate fermentation in the gut, decreased levels of SCFAs in patients with MDD open the doors for research into the impact of SCFAs on MDD. One of the ways that SCFAs prevent MDD is through the inhibition of pro-inflammatory cytokines. Increased presence of pro-inflammatory cytokines has already been implicated in the progression of MDD [71]. Since these cytokines can move freely during GD due to an increase in gut permeability, they are able to make their way past the BBB and infiltrate into the CNS, causing the progression of MDD [72,73]. SCFAs, specifically the butyrate, have a key role in upholding intestinal barrier integrity. Two SCFAs, namely butyrate and propionate, have been found to promote the production of Mucin-2, which is a protein that contributes to the integrity of the insoluble layer of the intestinal barrier [74]. Another way SCFAs can prevent MDD is through the modulation of epigenetic changes. Studies in mice models found that mice placed in stressful situations displayed decreased histone acetylation via higher levels of HDACs [75]. Studies in humans found increases in HDACs in patients with MDD and bipolar disorder [76,77]. Specifically, butyrate is important for inhibiting HDACs and promoting the production of brain-derived neurotrophic factor (BDNF) in mice models [65,77]. Lower levels of BDNF have been found in patients with MDD and other mood disorders [78]. Through SCFAs, the gut can modulate to improve the condition of this mental health disorder. So, SCFAs are key to treating mental health issues in patients with GD occurrence post SCI.

Another way GD can negatively impact mental health post SCI is through changes in production of neurotransmitters. It is believed that there are five phyla in the gut that are associated with the metabolism of tryptophan (Trp): Actinobacteria, Firmicutes, Proteobacteria, Bacteroidetes, and Fusobacteria [84]. Aside from production of serotonin (5-HT), Trp is crucial to the production of other signaling molecules such as indole-3-lactic acid (ILA), 3-Indolepropionic acid (IPA), tryptamine, 5-hydroxy indole acetic acid (5-HIAA), and more [85]. One study in mice models found that there was an elevated level of Trp in germ-free mice, but Trp levels returned to normal once bacteria were allowed to colonize the gut of the germ-free mice [86]. This change in Trp levels from the germ-free mice to the mice with regular bacterial composition suggests that Trp is susceptible to alterations in the gut bacterial composition. This is extremely concerning considering how important Trp is for 5-HT synthesis and how approximately 95% of 5-HT in the body is generated in the gut [87]. Studies in chronically stressed rats showed that treatment with *Lactobacillus helveticus* NS8 helped ameliorate anxiety and depressive-like behaviors in the rats while also increasing 5-HT levels [88]. The importance of 5-HT in functions like pain recognition and emotion makes it one of the very vital neurotransmitters to a well mental health state. Dysregulation of 5-HT has been linked to disorders like MDD, schizophrenia, and anxiety [89]. The mechanism by which 5-HT is produced in the gut is through enterochromaffin (EC) cells that send 5-HT to the afferent neurons in the gut mucous, triggering messaging to the CNS [87]. One study with irritable bowel syndrome (IBS) patients found that IBS patients had increased levels of *Ruminococcus torques* in the gut mucosa with noted higher levels of EC cells, suggesting that EC cells could also be influenced by gut bacterial composition [90]. A new possibility for treating psychiatric disorders is understanding the relationship between EC cells and GD since it has been found that mice with mutations promote depression and display low levels of EC cells [91].

Another neurotransmitter that has been shown to be influenced by the change in gut bacterial composition is dopamine (DA). Since DA is part of so many critical processes like emotion, learning, and motivation, changes in DA production have been implicated in MDD, compulsive disorders, anxiety disorders, and many more psychiatric ailments [92]. A study in mice showed that mice with their gut cleared with antibiotics had lower levels of tyrosine hydroxylase, an important enzyme in the rate-limiting step of DA synthesis [93]. Once the mice were off antibiotic treatment and able to return to normal gut bacterial composition, researchers noted a return to normal tyrosine hydroxylase levels. Studies have also found that the gut can influence the mesolimbic pathway, which is an important mechanism of DA synthesis and transportation in the brain. One study found that mice with social deficits had reduced gut diversity, but their gut diversity and behavior became normal after treatment with the *Lactobacillus reuteri* [94]. Researchers found that the mechanism by which behavior was restored was through *L. reuteri* inducing stronger long-term potentiation in neurons of the ventral tegmental area, a part of the mesolimbic pathway. Another study showed that the increased presence of bacteria from the families Ruminococcaceae and Lachnospiraceae decreased D2 receptors, a class of DA receptors, in the dorsal striatum of the alcohol-dependent rats [95]. Although the dorsal striatum is part of the basal ganglia, it still has close communication with the mesolimbic pathway [96]. Given the importance of DA in signaling for multiple psychiatric disorders, further understanding of how GD can influence mental health via DA is crucial to treatment of patients post SCI.

## 6. NLRP3 Inflammasome Activation and Mental Health Disorders

One mechanism by which NLRP3 inflammasome activation can affect mental health post SCI is through the maturation of pro-inflammatory cytokines. After a traumatic event or impact of a stressor, the immune system triggers the release of damage-associated molecular patterns (DAMPs), which are molecules that are released from the damaged or dying cells due to trauma or stress and activate the innate immune system by interacting with pattern recognition receptors (PRRs) [97]. DAMPs include multiple classes of molecules such as ATP, pro-inflammatory cytokines, and more. The release of DAMPs also triggers the release of PRRs. Among the PRRs, there are four different groups, and one of those groups is the NLR family, which is responsible for the formation of NLRP3 inflammasome [98]. After NLRP3 inflammasome activation, pro-inflammatory cytokines are primed to affect various processes across the body (Table 2) [99]. In a study with MDD patients, researchers analyzed blood serum of the patients and found elevated levels of the pro-inflammatory cytokine IL-1β [100]. Additionally, the amount of IL-1β present in the serum also correlated to the severity of MDD in the patients. Another study using the same methodology found similar results along with presence of the pro-inflammatory cytokine IL-18 in the MDD patients [101].

Another way by which NLRP3 inflammasome activation can induce depression is through the production of caspase-1. In a study with mice placed under chronic stress, the knockout mice with no caspase-1 mRNA displayed less anxiety and depressive-like behavior when they were compared to normal mice [7]. Researchers believed this was the result since the lack of caspase-1 would not result in the maturation of pro-inflammatory cytokines including IL-1β, all of which were implicated in mental health etiology. Although caspase-1 is implicated in the maturation of pro-inflammatory cytokines, there are other ways by which it can propagate psychiatric disorders. Another way caspase-1 impacts mental health is through modulation of the protein gasdermin D (GSDMD, an acronym from combination of gastro and dermato, referencing the two original locations for their most expression), which is responsible for forming extensive pores in cell membranes [102]. Caspase-1 cleaves the GSDMD N-terminal (GSDMD-NT), and GSDMD-NT forces itself into the cell membrane, creating a pore in the membrane [103,104]. Pro-inflammatory cytokines, ions, Rho family of small guanosine triphosphatases (Rho GTPases), and more can pass through the pores that are as small as 10 GSDMD-NT subunits [105]. One study in stress-induced mice found increased levels caspase-1 and GSDMD-NT in the hippocampus, causing the depressive-like behavior in the mice [106]. Also, the study found an increase in these proteins in the hippocampus suggesting an increase in pyroptosis in astrocytes. To test if these depressive symptoms were related to the elevated levels of GSDMD-NT, researchers tested stress-induced GSDMD knockout mice and noted decreased depressive behavior in the mice, suggesting that GSDMD cleavage could trigger depression.

**Table 2:** Mental health disorders and NLRP3 inflammasome activation.

Disorder	Model	Findings	References
Depression	Humans diagnosed with MDD via the DSM-5	Patients that received treatment with anti-depressants reported significantly lower levels of NLRP3 and caspase-1 mRNA than the patients that did not receive anti-depressant treatment.	[107]
Bipolar disorder	Post-mortem examination of the frontal lobes of people diagnosed with bipolar disorder	Frontal lobe samples of patients diagnosed with bipolar disorder had higher levels of NLRP3, ASC, caspase-1, and pro-inflammatory cytokines than samples from the control group.	[108]
Schizophrenia	Humans with schizophrenia whose symptoms were measured via the Scale for the Assessment of Positive Symptoms (SAPS) and Scale for the Assessment of Negative Symptoms (SANS) scales	Peripheral blood mononuclear cell samples from patients with schizophrenia had higher levels of expression of NLRP3, the pro-NLRP3 receptor P2X7, and pro-inflammatory cytokines than samples from the control group.	[109]
Anxiety	Wild-type and *Nlrp3^−/−^* mice	After being exposed to electric shocks, the *Nlrp3^−/−^* mice displayed attenuated anxiety-like behaviors and greater fear memory extinction. However, after exposure to the electric shocks, the wild-type mice presented with increased formation of NLRP3 and ASC as well as greater anxiety-like behavior and decreased fear memory extinction.	[110]

## 7. Potential Treatment Strategies for Amelioration of GD and NLRP3 Inflammasome Activation in SCI

### 7.1. Fecal Microbiome Transplantation

Fecal microbiome transplantation (FMT) started out as a treatment for *Clostridium difficile* infections [111]. As a potential treatment strategy, FMT has proven to be phenomenally successful in prevention of GD post SCI as well. One study found that rats displayed GD and anxiety-like behaviors following induction of SCI at cervical region [112]. After treatment with FMT, rats with cervical SCI were reported to exhibit normalization of the gut bacterial composition and less anxiety-like behavior. One thing to note was that FMT did not have any significant impact on locomotor recovery in the SCI animals. One of the main key factors for the success of FMT is the state of the donor the sample is coming from. Another study in rats focused on treating GD post SCI with FMT samples from rats with either anxious or un-anxious behavior [113]. The study found that FMT that used samples from anxious rats was unsuccessful in normalizing GD post SCI and led to an increase in anxiety in the rats that were being treated. Another way FMT can treat psychiatric disorders is through the promotion of production of SCFAs. In a study with pediatric patients with *C. difficile* infection, researchers treated the patients with a 12-month long FMT regimen. After the treatment for 12 months, researchers noted significant increases in the production of the following SCFAs: isovaleric acid, acetic acid, and propionic acid [114]. Given how low levels of SCFAs are associated with MDD, FMT shows promises as a potential therapy against GD post SCI and warrants further research activities in SCI animal models as well as in SCI patients.

### 7.2. Phytochemicals

Phytochemicals, including flavonoids, are plant-derived secondary metabolites with medicinal values that have been implicated in combating obesity, diabetes, cardiovascular diseases, and many more disorders in the body, potentially through inhibition of NLRP3 inflammasome activation (Table 3) [115].

These phytochemicals show a great diversity in the mechanisms by which they target NLRP3 inflammasome. Some phytochemicals target the molecular construction of NLRP3 while others target the NLRP3 signaling pathway [122]. Furthermore, the use of virtual screening analysis presents an opportunity to quickly find and test phytochemicals that show promise in inhibiting NLRP3 inflammasome activation [123]. Various phytochemicals, especially flavonoids, show a lot of promise as inhibitors of GD and NLRP3 inflammasome activation in major neurodegenerative diseases [124,125]. However, a major consideration is the process of producing phytochemicals in the laboratories and approving them as valid treatment options in neurotrauma, including SCI. Since plant-based chemicals usually require more time and money to synthesize in the laboratories, further research should be conducted to ensure therapeutic efficacy of these phytochemicals [126].

### 7.3. Melatonin

Melatonin is known for its use in regulating sleep and the circadian rhythm, but it is also a powerful antioxidant [127]. One study in mice challenged with LPS found an increased production of pro-inflammatory cytokines, NLRP3, and ROS [128]. However, after treatment with melatonin, the mice were reported to have reduced levels of all the characteristics of NLRP3 inflammasome activation. Another study in human lens epithelial cells has noted the same effects when lens cells that are stimulated with white light emitting diode (LED) light, which normally triggers ROS production, but recorded significantly less NLRP3 inflammasome activation and ROS production after melatonin treatment [129]. In the treatment of SCI, melatonin has been found to inhibit NLRP3 inflammasome activation and ROS production through the nuclear factor E2-related factor 2/antioxidant responsive element (Nrf2/ARE) pathway, which has been implicated in antioxidant and anti-inflammatory processes [130]. One study in mice with SCI used ML385, an Nrf2 inhibitor, to see if the pathway was relevant to NLRP3 and ROS inhibition post SCI [131]. The group of mice treated with just melatonin recorded decreases in NLRP3 and ROS, but the group of mice treated with melatonin and ML385 did not show any significant effect in decreasing NLRP3 and ROS post SCI. Another study also found that treatment with melatonin post SCI in rats reversed neuron loss and increased water content at the spinal cord, suggesting melatonin might play a big role in neuroprotection and functional recovery post SCI [132]. Additionally, melatonin might also aid in reducing symptoms of psychiatric disorders since it was proven to be useful in the treatment of MDD, bipolar disorder, and attention deficit hyperactivity disorder (ADHD) [133]. Furthermore, melatonin plays a role in controlling the gut microbiome alteration post SCI. Studies show associations between treatment with melatonin and reduced intestinal permeability as well as better repair of the intestinal barrier [134]. Moreover, melatonin has been shown to improve neuroplasticity and axon regeneration after SCI by restraining oxidative stress, inflammation, and apoptosis [135]. Melatonin is already a widely studied and used molecule in the treatment of CNS diseases and injuries [136,137,138,139,140]. As such, it has a lot of promise for future development as a successful therapy for the inhibition of GD as well as of NLRP3 inflammasome activation post SCI.

### 7.4. Aids from AI Technology for Treatment of GD and NLRP3 Inflammasome in SCI

Advancement of AI technology looks highly promising for detecting precise location of SCI, developing drugs for its treatment, and predicting the outcomes in SCI preclinical models as well as in clinical settings [141,142,143,144,145]. Although the application of AI technology is still relatively a new development in the field of medicine, it has surprisingly moved from the promise to the practice of aiding care for mental health disorders as well [146,147,148]. In fact, AI has been heralded as a game-changer in mental health care, with its potential use as a complementary tool rather than a replacement for involvement of humans in mental health care can be transformative in enhancing diagnostics, treatments, and monitoring for mental health disorders [149,150,151].

The exciting news is that there are already several ways that AI can aid in treating GD and NLRP3 inflammasome activation in CNS disorders including SCI. One advancement is a non-invasive AI-guided sensor that tracks the gut microbiome in the various stages of Parkinson’s disease (PD). In a PD model, europium-based nanoparticles are orally administered and track changes in the gut through sensors [152]. From there, AI receives data and makes predictions about how the changes in the gut relate to the progression of PD. Although this has only been assessed in PD, the mechanism of application of AI shows the promise as an easy way to track gut bacterial composition and have AI make predictions about the state of the gut post SCI. Another application of AI is using machine learning to understand the efficacy of drugs that are being used to treat gut disorders. One study proposed the use of a machine learning model that used prior research on drug-gut interactions to determine how effective drugs that were in development could be [153]. Although this use of AI sounds promising, it depends on the quality and quantity of research that can be accessed. Without adequate and correct research, AI is prone to making skewed predictions [154]. As such, it is important that there are accessible and up-to-date data repositories as well as set rules for AI to use when making predictions [155]. One way that AI technology is being used to target NLRP3 inflammasome is through AI-guided peptide design. AI cross references chemical features of novel peptides with the possible effects on NLRP3 through molecular simulations. Through the simulations, AI was able to provide a picture of how the novel peptide would interact with the various components of NLRP3 inflammasome [156]. Another system that focuses on machine learning has already developed a promising molecule called CSC-6 that has been shown to block ASC creation during NLRP3 inflammasome formation [157].

## 8. Conclusions and Future Directions

The concept that our normal gut microbiome is connected to our normal mental health has been proposed a long time ago and proven conclusively with recent research [158,159]. It is not astonishing anymore that alteration in gut microbiome causing the onset of GD following induction of a traumatic SCI is connected to the manifestation of mental health disorders [160,161,162]. We now know evidently that a traumatic SCI has not only economic impacts but also mental health impacts, and both problems require further research for devising proper strategies for improving the overall wellbeing of the SCI patients [163,164,165]. As far as we have been able to find, our present article is the first to propose a comprehensive link starting with a traumatic SCI, which directly triggers the onset of GD and the activation of NLRP3 inflammasome contributing together or independently to the development of mental health disorders, thus weaving a complex network of interactions originating from a traumatic SCI. A further understanding of the molecular mechanisms of this complex network is expected to promote therapeutic strategies for targeting the factors for improving mental health disorders in SCI patients. However, it needs to be noted again that a strategy for restoring composition of gut microbiome is effective only in improving mental health status and it is not yet a magic bullet in restoring motor function after a traumatic SCI.

Post SCI, there are many harmful effects around the body in terms of secondary injury effects [166,167,168,169,170]. Among those, the onset of GD and the activation of NLRP3 inflammasome stand out as the major contributors to the mental health disorders in SCI. Given the prevalence of mental health disorders post SCI, it is of utmost importance to understand how these disorders could be influenced by development of GD and activation of NLRP3 inflammasome. Several potential therapies for these secondary injury effects as a means of treating mental health disorders in SCI have also been discussed in this article. Additionally, the emergence of AI technology and its different systems has opened new avenues for the prevention of GD and NLRP3 inflammasome activation in SCI. While a lot about GD and NLRP3 inflammasome has been investigated, there still needs to be more done to understand the mechanisms of how GD and NLRP3 inflammasome initiate and aggravate mental health disorders, especially in relation to induction of SCI. If there is a clearer understanding of these mechanisms, then it will be lot easier to develop treatments for mental health disorders in the context of SCI and its secondary injury effects.

## Figures and Tables

**Table 1:** GD and mental health disorders.

Mental Health Disorder	Model	Findings	References
Anxiety	Humans categorized into anxious and non-anxious groups via the Beck Anxiety Scale	Patients in the non-anxious group had better diversity and spread of gut bacteria than patients in the anxious group. Patients in the anxious group had fewer SCFA-producing bacteria.	[79]
Depression	Sprague-Dawley rats and Long-Evan rats classified into either vulnerable or resilient groups	Fecal transplants from the vulnerable group to rats in the resilient group showed greater depressive-like symptoms, higher BBB permeability, and more pro-inflammatory cytokines than rats treated with fecal transplants from the control group.	[80]
Schizophrenia	Humans diagnosed with schizophrenia via the DSM-5	Patients in the schizophrenia group presented with markers for high levels of bacterial translocation with LPS binding protein (LBP).	[81]
Bipolar disorder	Humans diagnosed with bipolar disorder via the DSM-4	Patients in the bipolar disorder group had lower levels of bacteria from the genus *Faecalibacterium*, the producer of SCFAs.	[82]
Anorexia nervosa	Humans diagnosed with anorexia nervosa via the DSM-5	Patients in the anorexia nervosa group presented with lower levels of bacteria from the genus *Faecalibacterium*, the producer of SCFAs.	[83]

DSM-5 = Diagnostic and Statistical Manual of Mental Disorders, 5th Edition.

**Table 3:** Phytochemicals for inhibition of NLRP3 inflammasome activation.

Phytochemical	Model	Effect on NLRP3	Mechanism	References
Curcumin	C57BL/6 mice, LPS-primed macrophages	Suppression of caspase-1 and activation IL-1β maturation	Down-regulation of ERK signaling and inhibition of K^+^ efflux	[116]
Polydatin	Sprague-Dawley rats with SCI, BV2 mouse microglia	Reduced activation of NLRP3	Suppression of the NF-κB pathway	[117]
Tannic acid	Bone marrow derived macrophages from wild-type mice	Reduced activation of NLRP3 and IL-1β production	Suppression of the NF-κB pathway	[118]
Cardamonin	Human chondrocyte line	Suppression of NLRP3 and decreased presence of ASC and caspase-1	Interference of Nrf2/NQO1 signaling pathway	[119]
Oridonin	C57BL/6 mice, LPS-primed peripheral blood mononuclear cells	Inhibition of caspase-1 activation and IL-1β release	Blockage of interactions between NLRP3 and NEK7	[120]
Crocin	Monosodium urate-induced mice peritoneal macrophages	Inhibits pyroptosis caused by NLRP3-triggered cleavage of GSDMD	Blockage of production of mitochondrial ROS	[121]
